# Associations between maternal and infant selenium status and child growth in a birth cohort from Dhaka, Bangladesh

**DOI:** 10.1017/S0007114523000739

**Published:** 2023-11-14

**Authors:** Rukshan Mehta, Christine Krupa, Tahmeed Ahmed, Davidson H. Hamer, Abdullah Al Mahmud

**Affiliations:** 1 Centre for Global Child Health, The Hospital for Sick Children, Toronto, Canada; 2 Department of Nutritional Sciences, Faculty of Medicine, University of Toronto, Toronto, Canada; 3 Nutrition and Clinical Services Division, International Centre for Diarrhoeal Disease Research, Bangladesh (icddr,b), Dhaka, Bangladesh; 4 Department of Global Health, Boston University School of Public Health, Boston, MA, USA; 5 Section of Infectious Diseases, Boston University School of Medicine, Boston, MA, USA; 6 Gerald J. and Dorothy R. Friedman School of Nutrition Science and Policy, Tufts University, Boston, MA, USA

**Keywords:** Selenium, Linear growth, Whole blood selenium, Selenoprotein P, Serum selenium

## Abstract

Deficiency of essential trace element, Se, has been implicated in adverse birth outcomes and in child linear growth because of its important role in redox biology and associated antioxidant effects. We used data from a randomised controlled trial conducted among a cohort of pregnant and lactating women in Dhaka, Bangladesh to examine associations between Se biomarkers in whole blood (WBSe), serum and selenoprotein P (SEPP1) in maternal delivery and venous cord (VC) blood. Associations between Se biomarkers, birth weight and infant growth outcomes (age-adjusted length, weight, head circumference and weight-for-length z-scores) at birth, 1 and 2 years of age were examined using regression analyses. WB and serum Se were negatively associated with birth weight (adjusted *β*, 95 % CI, WBSe delivery: −26·6 (–44·3, −8·9); WBSe VC: −19·6 (–33·0, −6·1)); however, delivery SEPP1 levels (adjusted *β*: −37·5 (–73·0, −2·0)) and VC blood (adjusted *β*: 82·3 (30·0, 134·7)) showed inconsistent and opposite associations with birth weight. Positive associations for SEPP1 VC suggest preferential transfer from mother to fetus. We found small associations between infant growth and WBSe VC (length-for-age z-score *β*, 95 % CI, at birth: −0·05 (–0·1, −0·01)); 12 months (*β*: −0·05 (–0·08, −0·007)). Weight-for-age z-score also showed weak negative associations with delivery WBSe (at birth: −0·07 (–0·1, −0·02); 12 -months: −0·05 (–0·1, −0·005)) and in WBSe VC (at birth: −0·05 (–0·08, −0·02); 12 months: −0·05 (–0·09, −0·004)). Given the fine balance between essential nutritional and toxic properties of Se, it is possible that WB and serum Se may negatively impact growth outcomes, both in utero and postpartum.

## Graphical abstract

**Figure:**
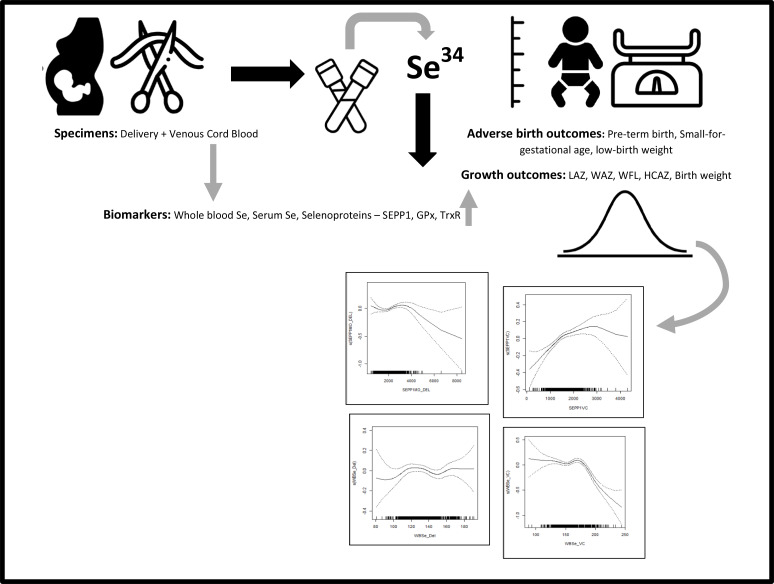

Linear growth, an important indicator of a child’s present and future health, is influenced by a complex interaction of environmental factors including intra-uterine growth restriction, household socio-economic status, parental education levels, inadequate maternal and child nutrition and frequent infections^(1,2)^. Global estimates (most recently from 2019) suggest that over 149 million children under the age of 5 are stunted^(3)^. According to the 2017–2018 Bangladesh Demographic and Health Survey, indicators of malnutrition among children under 5 years of age declined between 2007 and 2018, with the most recent prevalence estimates for stunting in children at 31 %, 22 % for underweight and 8 % for wasting^(4)^.

Se is an essential trace element that is known for its role in redox biology^(5)^. Due to adaptive changes in Se metabolism that occur during pregnancy, requirements are higher given its role in fetal growth, which manifests as lower maternal concentrations of blood Se^(6)^. Concentrations of this trace element are known to fall during pregnancy, with lowest levels observed at delivery^(7,8)^. Levels are lower because of increased fetal demands and alterations in intestinal absorption or renal handling^(7,8)^. Reduced Se concentrations can have an adverse impact on antioxidant selenoproteins, which compromise protection against placental oxidative stress during pregnancy, negatively impacting fetal growth^(7)^.

Se is known to play a protective role in many birth outcomes^(9)^. A recent meta-analysis found that umbilical cord blood Se concentrations (*r* 0·08, 95 % CI 0·01, 0·16) are correlated with birth weight, although similar results were not observed for associations between maternal blood Se and birth weight^(6)^. Other studies have demonstrated significant relationships between low birth weight (LBW) and lower Se concentrations^(6)^. Mechanisms for the associations between birth weight and Se remain unclear; however, its antioxidant properties play an important role in placental development and oxidative balance^(6)^. Lower Se levels are likely associated with reduced function of glutathione peroxidase, an antioxidant enzyme involved in protecting the placenta and fetus from oxidative stress^(5,10)^. In a similar manner, SEPP1 (selenoprotein P) is a selenoenzyme glycoprotein that protects against adverse pregnancy outcomes which can have an impact on fetal growth^(5,10)^. Se levels may also be correlated with small-for-gestational age (SGA) status; mechanistically, these associations may function via reductions in glucose levels which subsequently impact fetal weight^(11)^. Lower placental glutathione peroxidase activity has been observed in women with SGA compared with controls^(12)^. Due to the effects of lowered Se levels and decreased glutathione peroxidase activity during pregnancy, placental antioxidant defence may be reduced which in turn may impact fetal growth, resulting in adverse birth outcomes including growth restriction and SGA^(12)^. A comprehensive schematic of placental to fetal transfer of various selenometabolites and Se metabolism in the human body is presented in online Supplementary Fig. 1.

In addition to the effects of Se on birth weight and birth outcomes, this micronutrient also plays an essential role in bone growth and development. Se inadequacy has been implicated in growth faltering and changes in bone metabolism which can impact bone and cartilage development^(13–15)^. Expression of selenoproteins by bone cells can contribute to protection against oxidative stress in the microenvironment of the bone^(15)^. Se deficiency impacts bone formation and resorption, whereby the effects of Se on bone are mediated through reduced antioxidative capacity, accumulation of reactive oxygen species, reduced osteoclast activation and osteoblast differentiation^(16)^. Furthermore, Se deficiency is known to impact cartilage and can reduce bone mineral content, as described in human and animal studies^(13,14,16)^.

Se metabolism and use in the bone are exclusively associated with selenoproteins. A total of nine selenoproteins have been identified in human fetal osteoblasts where they play a role in bone metabolism^(15)^. SEPP1 is the primary transporter and storage selenoprotein from the liver to bones, where the Lrp8 receptor facilitates SEPP1-mediated Se uptake, particularly under conditions of Se deficiency^(17,18)^. Se therefore plays an important role in bone physiology and is tightly regulated by SEPP1 receptor, apolipoprotein receptor 2. Expression of this receptor is significantly influenced by SEPP1 levels in a negative feedback loop^(18)^. Genetic ablation of SEPP1 has been shown to reduce serum Se concentrations by 25-fold but only reduces bone Se levels by 2·5-fold, suggesting a protective effect^(18)^.

Studies have examined associations between Se levels in mother–infant dyads and growth outcomes at birth and in the postnatal period^(6,9,19)^. Given the mixed body of evidence on the association of Se with birth weight and infant growth outcomes, we sought to understand whether Se status measured in maternal and infant whole blood (WBSe), serum (serum Se) and a biomarker of functional status, SEPP1, at delivery and in venous cord (VC) blood is correlated with infant birth weight, growth outcomes (length-for-age z-score (LAZ), weight-for-age z-score, weight-for-length z-score and head circumference-for-age z-score) at birth, in infancy and up to 2 years of age. Our study will add to a growing body of evidence on the role Se may play in in utero and postnatal infant growth and development.

## Methods

### Study area and subjects

We used observational Se biomarker data collected as part of double-blinded, dose-varying, placebo-controlled, randomised trial of the effect of maternal vitamin D_3_ supplementation during pregnancy and lactation conducted in Dhaka, Bangladesh for this secondary analysis. Details of the parent study (NCT01924013) are described elsewhere^(20)^. Briefly, a total of 1300 women, 18 years of age and older, with uncomplicated singleton pregnancies were enrolled between 17 and 24 weeks of gestation and randomised into one of five treatment groups (treatment group A = 0 μg/week during pregnancy and postpartum, treatment group B = 4200 μg/week during pregnancy and placebo postpartum, treatment group C = 16 800 μg/week during pregnancy and placebo postpartum, treatment group D = 28 000 μg/week during pregnancy and placebo postpartum and treatment group E = 28 000 μg/week during pregnancy and postpartum).

Exclusion criteria included high risk pregnancies, women who had pre-existing medical conditions or those who were taking medications that could put them at risk of vitamin D sensitivity, altered vitamin D metabolism and hypercalcaemia. Additional details are provided elsewhere^(20)^.

Inclusion of participant samples for this secondary analysis was based on availability of at least one measurement of Se and/or selenoproteins at delivery in mothers or in VC blood of neonates. Infants for whom length and weight were not measured at birth or 12 months were excluded.

Se was measured as part of a panel of metals and minerals for which the primary study intent was to examine effects of vitamin D supplementation on heavy metal concentrations^(21)^. Samples selected for Se quantification were not related to vitamin D treatment group, and seasonal imbalances in specimen collection were observed for those selected to be a part of the sub-study^(21)^. Additional socio-demographic characteristics of study participants were recorded using standardised data collection forms.

### Blood collection

Maternal and VC blood samples were collected at delivery by trained phlebotomists. Trace metal-free EDTA-coated tubes were used to draw maternal samples, and whole-blood aliquots were drawn prior to centrifugation. Cord blood was collected within 30 min of delivery from a site on the umbilical cord attached to the placenta after wiping maternal blood away with dry gauze. The umbilical vein was cannulated to collect blood into a lavender top EDTA tube. Whole blood was stored at −70°C or colder.

### Measurement of selenium biomarkers

Whole blood and serum Se were measured at the US Centers for Disease Control and Prevention (CDC). WBSe was measured at delivery and in VC blood using inductively coupled plasma MS^(22)^. The lower limit of detection for this assay was 24·5 µg/l. Serum Se was measured at delivery using inductively coupled plasma-dynamic reaction cell-MS as described by Caldwell^(23)^. The lower limit of detection was 4·5 µg/l. No dilution factors were applied for these assays. Plasma SEPP1 was measured at delivery and in VC blood using a Selenotest ELISA colorimetric assay (STE, InVivo BioTech Services) performed at the Hospital for Sick Children. The lower limit of detection for the assay was 267·3 µg/l, with a 33-fold dilution factor applied and intra and inter-assay CV measured at 4·5 and 10·1 %, respectively. At least one Se biomarker was measured in a sub-sample of 1021 specimens collected from mothers or infants for inclusion in this study^(21)^.

### Outcome assessment

Details of outcome assessment have been elaborated on elsewhere^(20)^. In brief, head circumference, length and weight were measured by trained professionals using standardised procedures adapted from the INTERGROWTH-21st (International Fetal and Newborn Growth Consortium for the 21st Century) Project^(24)^. Infant length was measured using a ShorrBoard. Infant weight was measured using a digital scale. Length and weight were measured at birth, during visits at 1–2 months, 3, 6, 9, 12, 15, 21 and 24 months of age. For the purposes of our study, growth outcomes were assessed at birth, 12 and 24 months of age.

Two independent personnel measured each child, and repeated measurements were taken. Measurements that differed between the two personnel by 7 mm for length or 50 g for weight were taken again. Means of the final pairs were used in further analysis. Inter-rater reliability was high. Length, weight and weight-for-length, BMI and head circumference were converted to age standardised z-scores using INTERGROWTH-21st standards for newborn size, postnatal growth standards for preterm infants to 64 weeks of postmenstrual age (weight, length and head circumference only) or WHO child growth standards, as appropriate^(24)^. All outcome variables were modelled continuously. We also examined three birth outcomes, namely LBW, classified as birth weight < 2500 g, and SGA, defined as birth weight below the 10th percentile of gestational age- and sex-matched healthy reference population, as dichotomous outcomes^(25)^. Preterm birth (PTB) was defined as gestational age at birth less than 37 weeks^(26)^.

### Covariates

Covariates adjusted for in models included infant characteristics, such as sex, gestational age at birth and season of birth, and maternal characteristics including age, education, height, BMI, gravidity, vitamin D treatment group, daily protein intake (kg), urinary cotinine levels, maternal smoking and tobacco use during pregnancy, C-reactive protein concentrations at delivery and household asset index.

Gestational age was estimated at the time of enrolment using a combination of the last menstrual period and ultrasound. Smoking status was described using urinary cotinine levels, where women with cotinine ≤ 50 ng/ml were classified as non-smokers and women with levels > 50 ng/ml were classified as smokers^(27)^. An asset index was derived using principal component analysis on baseline household characteristic data collected on ownership of various assets, where each participant was assigned an asset score (lower scores reflect less wealth)^(20)^. The asset index was categorised into quintiles.

### Statistical analysis

All analyses were conducted using SAS 9.4 (SAS Institute). A *P*-value < 0·05 was considered statistically significant. Descriptive statistics were generated for all outcome variables (growth indices) and Se biomarkers as means and standard deviations, median (inter-quartile range) depending on the shape of variable distributions. Bivariate correlations between all Se biomarkers were evaluated using Spearman correlation coefficients.

**Fig. 1.**
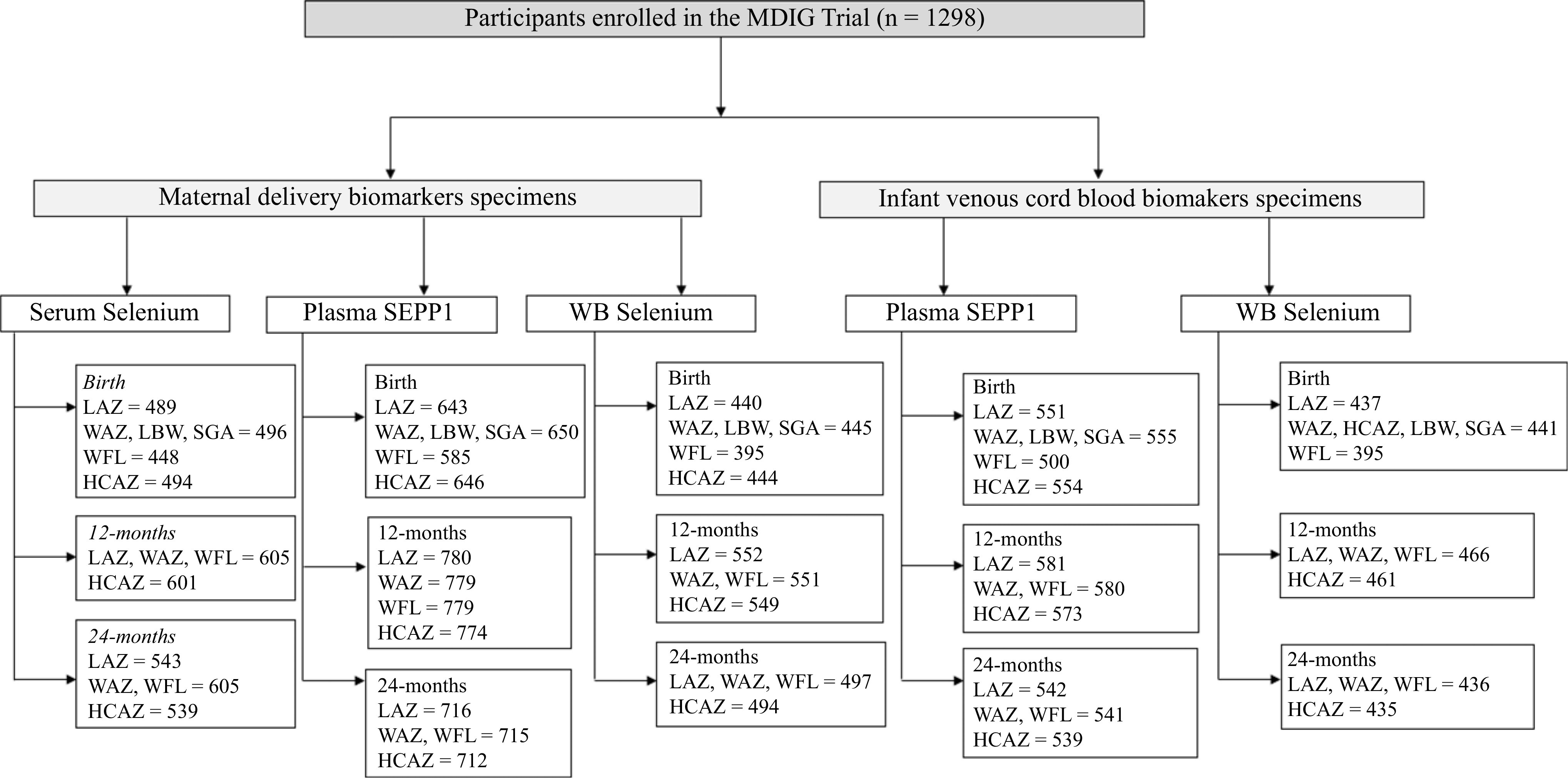
Sample size chart for maternal and cord Se biomarkers included in the analysis of associations with growth outcomes. LAZ, length-for-age z-score; WAZ, weight-for-age z-score; WFL, weight-for-length; HCAZ, head circumference for age z-score; LBW, low birth weight; SGA, small for gestational age.

We assessed normality of all variables in this analysis using histograms and kernel density plots in order to determine their distribution. Outliers were flagged and examined to determine their biological plausibility, using box plots. Data for this secondary analysis were restricted to those participants with any one of the Se biomarkers in maternal delivery or VC specimens for which a growth measure was also available, as presented in Fig. 1.

We used linear regression and generated bivariate and multivariable models adjusting for covariates after generating directed acyclic graphs to account for hypothesised confounding and biological plausibility of associations. Biomarkers were scaled by a unit of 10 for whole blood and serum Se and unit of 1000 for SEPP1. Sensitivity analyses were conducted after including urinary cotinine as a covariate in adjusted models.

We also examined associations between selenoproteins glutathione peroxidase and thioredoxin reductase measured in maternal plasma at mid-gestation (17–24 weeks) and delivery and growth outcomes using linear regression, associations with birth weight, specifically (presented in online Supplementary Tables 1 and 2). Multicollinearity between predictors and covariates in models was assessed using variance inflation factors, and tolerance and collinearity using ‘proc reg’.

#### Selenium biomarkers and birth outcomes

Descriptive statistics for *n* (%) of children who were SGA, LBW and PTB in this sample were also examined. We conducted additional analyses to examine associations between SGA, LBW and PTB as dichotomous outcomes using modified Poisson regression with robust standard errors to estimate risk ratios for associations between birth outcomes and Se biomarker concentrations in unadjusted and adjusted models.

#### Mediation analysis

In addition to linear models for growth outcomes, we conducted mediation analyses using observed variables to explore potential causal pathways for associations between cord WBSe and SEPP1 and weight at birth (birth weight and weight-for-age z-scores (WAZ)) and 1 year of age. We hypothesised that SEPP1 may mediate the association between WBSe in VC blood and WAZ/birth weight. We modelled both endogenous (outcome) and exogenous (predictor) variables continuously in structural equation modelling path analysis^(28)^. Mediation path analyses were conducted using ‘proc calis’ in SAS 9.4 and verified in RStudio using the ‘sem’ function. We examined direct, indirect and total effects. Model fit was assessed using fit statistics including standardized root mean square residual (SRMR) (< 0·08), root mean square error of approximation (RMSEA) (< 0·08), *χ* ^2^ (*P*-value > 0·05), comparative fit index (CFI) (≥ 0·90), goodness of fit index (GFI) (≥ 0·95) and Tucker-Lewis index (TLI) (≥ 0·95) and benchmarked based on established criterion^(29)^. We did not adjust for covariates in path analyses, and results are presented as standardised estimates.

#### Longitudinal models and generalised additive models

We applied a gate-keeper approach to assess longitudinal associations between growth outcomes and Se biomarkers based on significant associations observed in cross-sectional linear analyses at ages 0, 1 and 2 years. We used unadjusted multilevel models of change (‘proc mixed’) to assess whether relevant Se biomarkers in VC blood predicted a change in growth-z-scores between 0 and 3 years based on the availability of anthropometric data. We also used generalised additive models (‘proc gam’) to examine smoothing splines for predicted associations between Se biomarkers and relevant measures of growth.

## Results

### Participant characteristics

A detailed description of analytical sample sizes across growth outcomes in our study of mother–infant dyads is presented in Fig. 1, for three timepoints, namely birth, 12 months and 24 months of age. Given the distribution of observations with available Se biomarkers, our sample sizes ranged between 435 and 780 for these analyses across growth outcomes. On average, mothers were 23 years of age (sd 4), a majority were educated at a secondary school level or higher, median gestational age at birth was 39 weeks (inter-quartile range 2) and mean birth weight across our sample was 2724 (sd 354) g, with close to 46 % of children categorised as SGA, approximately 25 % were LBW (< 2500 g) and 8 % were born before 37 weeks of gestation (Table 1). A small proportion of our sample was below the threshold (< 100 µg/l) for WBSe in maternal delivery and VC specimens (2·6 % and 0·2 %, respectively), and 83·4 % of specimens were below threshold for serum Se at delivery (< 80 µg/l) and 0·2 % of the sample was classified as deficient (< 30 µg/l). Se biomarker concentrations are presented in Table 2.

**Table 1.** Socio-demographic characteristics and Se status of women and children in the study sample (Mean values and standard deviations; medians and inter-quartile ranges; numbers and percentages)

Maternal and household characteristics*,†	Maternal delivery specimens						Infant venous cord specimens			
	Whole blood Se		Serum Se		SEPP1		Whole blood Se		SEPP1	
	Mean	sd	Mean	sd	Mean	sd	Mean	sd	Mean	sd
*n*	467		517		677		448		562	
Age (years)	23	4	23	4	23	4	23	4	23	4
Gravidity	2	1	2	1	2	1	2	1	2	1
Height (cm)	151	6	151	5	151	5	151	6	151	6
Gestational age at delivery (in weeks)										
Median	39·1		39·1		39·1		39·1		39·3	
IQR	1·7		1·9		1·7		1·7		1·9	
Weight at delivery (kg)	61	10	61	10	61	10	61	10	61	10
BMI (kg/m^2^)	24	4	24	4	24	4	24	4	24	4

	*n*	%	*n*	%	*n*	%	*n*	%	*n*	%
Ever smoked during pregnancy	4	0·9	3	0·6	4	0·6	2	0·5	4	0·7
Ever used tobacco during pregnancy	179	38	191	37	235	35	161	36	195	35
Urinary cotinine (> 50 ng/ml)	18	4	16	3	16	3	13	3	13	2
Daily protein intake (kg/d)										
Mean	0·8		0·9		0·9		0·9		0·9	
sd	0·3		0·3		0·4		0·4		0·4	
Household asset index quintile, *n* (%)‡										
1	92	20	94	18	120	18	85	19	108	19
2	94	20	99	19	132	20	81	18	102	18
3	95	20	115	22	144	21	103	23	123	22
4	96	21	103	20	139	21	92	21	113	20
5	88	19	102	20	139	21	84	19	113	20
Maternal education, *n* (%)†										
No education	20	4	25	5	27	4	13	3	19	3
Primary incomplete	96	21	104	20	127	19	88	20	105	19
Primary complete	64	14	67	13	93	14	61	14	76	14
Secondary incomplete	175	37	194	38	267	39	168	38	214	38
Secondary complete	112	24	127	25	163	24	118	26	148	26
Season, *n* (%)†										
Spring	–		67	13	107	16	–		88	16
Summer	157	35	160	32	183	28	168	38	172	31
Fall	175	39	173	34	220	33	168	38	182	32
Winter	120	27	106	21	151	23	111	25	119	21
Infant characteristics‡										
Sex (male), *n* (%)	221	49	236	48	314	48	224	51	277	50
Gestational age at birth (weeks)										
*n*	452		506		661		447		561	
Median	39		39		39		39		39	
IQR	2		2		2		2		2	
Birth outcomes	Mean		sd		Mean		sd		Mean	
*n*	445		496		650		441		555	
Birth weight (g)	2706	330	2721	346	2721	350	2742	328	2747	339
Small-for-gestational age										
*n*	224		233		302		202		253	
%	50		47		46		46		46	
Low birth weight (< 2500 g)										
*n*	124		131		169		105		130	
%	28		26		26		24		23	
Preterm birth (gestational age < 37 weeks)*										
*n*	39		53		64		26		33	
%	8		10		9		6		6	
*n*	440		489		643		437		551	
Length-for-age z-score†, §	–0·97	0·99	–0·88	0·98	–0·88	0·99	–0·88	0·99	–0·86	1·00
*n*	445		496		650		441		555	
Weight-for-age z-score†, §	–1·24	0·82	–1·17	0·84	–1·19	0·86	–1·14	0·81	–1·15	0·83
*n*	395		448		585		395		500	
Weight-for-length z-score†, §	–0·73	0·94	–0·72	0·93	–0·73	0·95	–0·65	0·92	–0·67	0·93
*n*	444		494		646		441		554	
Head circumference-for-age z-score†, §	–0·67	0·94	–0·62	0·94	–0·62	0·95	–0·59	0·92	–0·59	0·94
12-month growth outcomes										
*n*	552		605		780		466		581	
Length-for-age z-score†,§	–1·01	1·01	–0·99	1·04	–0·99	1·06	–0·89	1·00	–0·87	1·02
*n*	551		605		779		466		580	
Weight-for-age z-score†,§	–0·88	1·06	–0·88	1·09	–0·87	1·10	–0·74	1·08	–0·78	1·10
*n*	551		605		779		466		580	
Weight-for-length z-score†,§	–0·52	1·01	–0·53	1·03	–0·52	1·04	–0·42	1·04	–0·48	1·06
*n*	549		601		774		461		573	
Head circumference-for-age z-score†,§	–1·23	0·96	–1·18	0·99	–1·19	0·98	–1·14	0·90	–1·16	0·99
24-month growth outcomes										
*n*	497		543		716		436		542	
Length-for-age z-score†,§	–1·28	1·04	–1·32	1·08	–1·32	1·09	–1·22	1·05	–1·22	1·07
*n*	497		542		715		436		541	
Weight-for-age z-score†,§	–1·17	1·16	–1·21	1·17	–1·20	1·16	–1·07	1·18	–1·11	1·20
*n*	497		542		715		436		541	
Weight-for-length z-score†,§	–0·72	1·09	–0·74	1·09	–0·71	1·07	–0·61	1·12	–0·67	1·14
*n*	497		539		712		435		539	
Head circumference-for-age z-score†,§	–1·30	0·94	–1·28	0·97	–1·30	0·96	–1·25	0·88	–1·28	0·99
Vitamin D treatment group	*n*	%	*n*	%	*n*	%	*n*	%	*n*	%
Placebo control	133	17	67	15	2171	788	155	21	1481	486
4200 μg/week	133	17	69	11	2162	761	155	24	1472	496
16 800 μg/week	136	20	70	12	2161	666	161	20	1597	592
28 000 μg/week pregnancy only	135	19	67	14	2087	740	163	22	1550	461
28 000 μg/week pregnancy & postpartum	131	15	69	12	2138	605	162	22	1566	553

SEPP1, selenoprotein P.

* Whole blood Se < threshold (< 100 µg/l), serum Se < threshold (< 80 µg/l), serum Se deficiency (< 30 µg/l), no thresholds exist for SEPP.

† Biomarker concentrations are un-scaled, sample sizes represent age at birth (up to 1-d postpartum) and maximum observations with Se biomarkers available.

‡ Sample sizes do not correspond to totals for biomarker concentrations, observations without corresponding Se biomarkers are excluded.

§ All values are presented as mean (sd) unless indicated otherwise.

**Table 2.** Se biomarker concentrations at delivery and in infant venous cord blood (numbers and percentages; mean values and standard deviations)

Biomarkers*	Maternal delivery specimens						Infant venous cord specimens			
	WBSe		Serum Se		SEPP1		WBSe		SEPP1	
	*n*	%	*n*	%	*n*	%	*n*	%	*n*	%
n*	467		517		677		448		562	
Se biomarker concentration (µg/l)										
Mean	133·5		68·6		2144·0		159·2		1535·0	
sd	17·8		12·8		712·0		22·0		522·8	
Minimum, maximum (µg/l)	80·5, 190·6		4·5, 110·3		524·8, 4968·4		88·1, 233·4		117·2, 3800·6	
* *< Threshold*	12	2·6	431	83·4	–		1	0·2	–	
* *Deficient†	–		1	0·2	–		–		–	
C-reactive protein (CRP) maternal delivery plasma, *n*	437		509		672		417		535	
CRP maternal delivery plasma (mg/l)										
Median	9		8		8		17		8	
IQR	18		16		18		24		15	
CRP venous cord plasma, *n*	272		322		427		348		450	
CRP venous cord plasma (mg/l)										
Median	1		1		1		0·8		1	
IQR	1		0		0		0·4		0	
CRP venous cord plasma (detectable)	202	74	254	79	342	80	263	76	356	79

SEPP1, selenoprotein P; IQR, inter-quartile range.

* WBSe above threshold defined as concentrations ≥ 100 µg/land serum Se ≥ 80 µg/l.

† Deficiency defined as serum Se < 30 µg/l.

Spearman correlation coefficients for associations between delivery and VC Se biomarkers in our analyses are described in Table 3. We found small to moderate correlations between Se biomarkers at delivery and in VC blood, with the exception of SEPP1 and WBSe where associations were non-significant (WBSe Del and WBSe VC: *ρ* = −0·00001, *P* = 0·9; SEPP1 Del and WBSe VC: *ρ* = 0·03, *P* = 0·6) in both specimen types.

**Table 3.** Spearman correlation coefficients (*ρ*) for maternal delivery and venous cord Se biomarkers

	Maternal delivery specimens									Infant venous cord specimens					
	WBSe			Serum Se			SEPP1			WBSe			SEPP1		
	*n*	*ρ*	*P*	*n*	*ρ*	*P*	*n*	*ρ*	*P*	*n*	*ρ*	*P*	*n*	*ρ*	*P*
WBSe Del	447	1	–	376	0·6	< 0·0001	385	0·3	< 0·0001	332	0·5	< 0·0001	320	–0·00001	0·9
Serum Se Del	376	0·6	< 0·0001	497	1	–	487	0·4	< 0·0001	321	0·3	< 0·0001	389	0·1	0·02
SEPP1 Del	385	0·3	< 0·0001	487	0·4	< 0·0001	651	1	–	377	0·03	0·6	488	0·2	< 0·0001
WBSe VC	332	0·5	< 0·0001	321	0·3	< 0·0001	377	0·03	0·6	443	1	–	404	0·1	0·001
SEPP1 VC	320	–0·00001	0·9	389	0·1	0·02	488	0·2	< 0·0001	404	0·2	0·001	557	1	–

WBSe, whole blood selenium; SEPP1, selenoprotein P; VC, venous cord.

### Modelling analyses

Linear regression analyses are presented for unadjusted and adjusted associations between Se biomarkers and growth outcomes in Table 4.

**Table 4.** Linear and longitudinal regression analyses for associations between Se biomarkers and growth outcomes (95 % confidence intervals)

Outcome	Se Biomarker*	Timepoint	*n*	Unadjusted *β*	95 % CI	*P*	*n*	Adjusted *β* †,‡	95 % CI	*P*
Birth weight	WBSe delivery	Birth	445	–9·6	–27·2, 7·9	0·3	416	–26·6	–44·3, −8·9	0·003
	WBSe venous cord		441	–23·6	–37·4, −9·8	0·001	411	–19·6	–33·0, −6·1	0·005
	Serum Se delivery		496	–2·9	–26·8, 21·1	0·8	486	–16·4	–38·8, 6·0	0·2
	SEPP1 delivery		650	6·2	–31·9, 44·3	0·7	643	–37·5	–73·0, −2·0	0·04
	SEPP1 venous cord		555	43·3	–10·9, 97·5	0·1	527	82·3	30·0, 134·7	0·002
LAZ	WBSe delivery	Birth	440	–0·03	–0·1, 0·03	0·3	412	–0·05	–0·1, 0·002	0·06
		12 months	552	–0·006	–0·05, 0·04	0·8	521	–0·03	–0·08, 0·01	0·2
		24 months	497	0·002	–0·05, 0·05	0·9	468	–0·02	–0·07, 0·03	0·4
LAZ§	WBSe venous cord§	Birth	437	–0·06	–0·1, −0·01	0·009	407	–0·05	–0·1, −0·01	0·01
		12 months	466	–0·05	–0·09, −0·01	0·02	442	–0·05	–0·08, −0·01	0·02
		24 months	436	–0·05	–0·1, −0·01	0·01	409	–0·04	–0·08, 0·003	0·07
LAZ	Serum Se delivery	Birth	489	0·01	–0·06, 0·08	0·8	479	–0·03	–0·1, 0·04	0·4
		12 months	605	–0·01	–0·07, 0·04	0·6	597	–0·05	–0·1, 0·01	0·09
		24 months	543	–0·01	–0·08, 0·05	0·7	533	–0·05	–0·1, 0·01	0·1
LAZ	SEPP1 delivery	Birth	643	–0·01	–0·1, 0·1	0·9	636	–0·07	–0·2, 0·04	0·2
		12 months	780	0·03	–0·07, 0·1	0·6	775	–0·01	–0·1, 0·07	0·7
		24 months	716	0·03	–0·07, 0·1	0·5	709	–0·03	–0·1, 0·06	0·5
LAZ	SEPP1 venous cord	Birth	551	0·1	–0·02, 0·3	0·08	523	0·2	0·04, 0·4	0·01
		12 months	581	0·1	–0·03, 0·3	0·1	555	0·07	–0·09, 0·2	0·4
		24 months	542	0·2	–0·006, 0·3	0·06	517	0·1	–0·06, 0·3	0·2
WAZ	WBSe delivery	Birth	445	–0·04	–0·1, 0·001	0·06	416	–0·07	–0·1, −0·02	0·004
		12 months	551	–0·01	–0·06, 0·03	0·6	521	–0·05	–0·1, −0·005	0·03
		24 months	497	0·01	–0·04, 0·07	0·7	468	–0·02	–0·08, 0·03	0·5
WAZ	WBSe venous cord	Birth	441	–0·04	–0·1, −0·003	0·03	411	–0·05	–0·08, −0·02	0·004
		12 months	466	–0·04	–0·08, 0·003	0·07	442	–0·05	–0·09, −0·004	0·03
		24 months	436	–0·04	–0·09, 0·007	0·1	409	–0·03	–0·08, 0·02	0·3
WAZ	Serum Se delivery	Birth	496	–0·00002	–0·06, 0·06	0·99	486	–0·04	–0·09, 0·02	0·2
		12 months	605	–0·03	–0·09, 0·04	0·4	597	–0·07	–0·1, 0·01	0·02
		24 months	542	–0·008	–0·08, 0·06	0·8	532	–0·05	–0·1, 0·02	0·2
WAZ	SEPP1 delivery	Birth	650	–0·02	–0·1, 0·1	0·6	643	–0·09	–0·2, 0·003	0·06
		12 months	779	0·004	–0·09, 0·1	0·9	774	–0·06	–0·2, 0·04	0·2
		24 months	715	0·0003	–0·1, 0·1	0·9	708	–0·08	–0·2, 0·03	0·1
WAZ§	SEPP1 venous cord§	Birth	555	0·1	0·006, 0·3	0·04	555	0·1	0·006, 0·3	0·04
		12 months	580	0·2	0·002, 0·3	0·05	554	0·08	–0·09, 0·2	0·4
		24 months	541	0·2	0·04, 0·4	0·02	516	0·1	–0·07, 0·3	0·2
WFL	WBSe delivery	Birth	395	–0·02	–0·1, 0·03	0·5	370	–0·04	–0·1, 0·02	0·2
		12 months	551	–0·02	–0·1, 0·03	0·5	521	–0·05	–0·1, −0·005	0·03
		24 months	497	0·01	–0·04, 0·07	0·6	468	–0·01	–0·07, 0·04	0·6
WFL	WBSe venous cord	Birth	395	–0·008	–0·05, 0·03	0·7	369	–0·03	–0·07, 0·02	0·2
		12 -months	466	–0·02	–0·06, 0·02	0·3	442	–0·03	–0·07, 0·01	0·1
		24 months	436	–0·01	–0·06, 0·03	0·5	409	–0·01	–0·06, 0·04	0·7
WFL	Serum Se delivery	Birth	448	0·01	–0·06, 0·08	0·7	439	–0·02	–0·09, 0·05	0·6
		12 months	605	–0·03	–0·08, 0·03	0·4	597	–0·07	–0·1, −0·007	0·03
		24 months	542	–0·005	–0·07, 0·06	0·9	532	–0·03	–0·1, 0·03	0·3
WFL	SEPP1 delivery	Birth	585	–0·01	–0·1, 0·1	0·8	579	–0·06	–0·2, 0·06	0·3
		12 months	779	–0·02	–0·1, 0·07	0·7	774	–0·07	–0·2, 0·02	0·1
		24 months	715	–0·03	–0·1, 0·07	0·6	708	–0·09	–0·2, 0·02	0·1
WFL	SEPP1 venous cord	Birth	500	0·1	–0·1, 0·3	0·2	475	0·1	–0·02, 0·3	0·09
		12 months	580	0·1	–0·01, 0·3	0·07	554	0·06	–0·1, 0·2	0·5
		24 months	541	0·2	0·005, 0·4	0·04	516	0·1	–0·1, 0·3	0·3
HCAZ	WBSe delivery	Birth	444	–0·02	–0·07, 0·03	0·5	416	–0·04	–0·09, 0·01	0·1
		12 months	549	–0·02	–0·06, 0·03	0·4	518	–0·02	–0·07, 0·02	0·4
		24 months	494	–0·005	–0·05, 0·04	0·8	465	–0·008	–0·06, 0·04	0·7
HCAZ	WBSe venous cord	Birth	441	0·003	–0·04, 0·04	0·9	411	–0·002	–0·04, 0·04	0·9
		12 months	461	–0·02	–0·06, 0·01	0·2	437	–0·02	–0·06, 0·02	0·3
		24 months	435	–0·02	–0·06, 0·01	0·2	408	–0·02	–0·05, 0·02	0·5
HCAZ	Serum Se delivery	Birth	494	0·02	–0·05, 0·08	0·6	484	–0·01	–0·08, 0·05	0·7
		12 months	601	0·002	–0·05, 0·06	0·9	593	–0·005	–0·1, −0·05	0·9
		24 months	539	–0·02	–0·07, 0·04	0·6	529	–0·02	–0·08, 0·04	0·4
HCAZ	SEPP1 delivery	Birth	646	–0·1	–0·2, 0·04	0·2	639	–0·1	–0·2, −0·02	0·02
		12 months	774	0·02	–0·07, 0·1	0·7	769	–0·01	–0·1, 0·08	0·8
		24 -months	712	–0·03	–0·1, 0·06	0·6	705	–0·06	–0·2, 0·03	0·2
HCAZ	SEPP1 venous cord	Birth	554	0·04	–0·1, 0·2	0·6	526	0·07	–0·09, 0·2	0·4
		12 months	573	–0·02	–0·2, 0·1	0·8	547	–0·06	–0·2, 0·1	0·5
		24 months	539	–0·03	–0·2, 0·1	0·7	514	–0·06	–0·2, 0·1	0·5

WBSe, whole blood selenium; SEPP1, selenoprotein P; LAZ, length-for-age z-score; WAZ, weight-for-age z-score; WFL, weight-for-length; HCAZ, head circumference for age z-score; CRP, C-reactive protein.

* WBSe scaled to represent the change in anthropometric outcome for every 10 µg/l increase in WBSe. Serum Se scaled to represent the change in anthropometric outcome for every 10 µg/l increase in serum Se. SEPP1 scaled to represent the change in anthropometric outcome for every 1000 µg/l increase in SEPP1.

† Models adjusted for infant sex, gestational age at birth, season of birth, maternal age, maternal education, maternal height, maternal BMI, gravidity, vitamin D treatment group, delivery CRP, asset index quintiles, daily protein intake (kg), smoking and tobacco use during pregnancy.

‡ No multi-collinearity problem was detected in models – assessment was conducted after generating directed acyclic graphs based on conceptual frameworks developed *a priori*.

§ Longitudinal analysis only conducted where unadjusted linear regression associations were significant for at least two of three age points.

#### Whole blood selenium and growth outcomes

We examined associations between WBSe and birth weight among infants in our sample. We found negative associations between delivery WBSe and birth weight in unadjusted models, where per scaled unit increased in WBSe, birth weight decreased by 10 g, although results were not statistically significant.

Likewise, we found null associations (risk ratio: 1·03, 95 % CI: 0·96, 1·1) for LBW per unit (g) increase in maternal delivery WBSe in unadjusted models, with increased risk (12–15 %) after accounting for confounders.

In line with our findings for maternal delivery WBSe, we found estimates for associations between VC WBSe and birth weight to be significant with effect estimates suggesting a decrease in birth weight of between 24 and 27 g per unit increase in Se, between unadjusted and adjusted models. Results for risk of LBW were in concordance with our findings for birth weight, with a 6–11 % increased risk of the outcome per unit increase in WBSe in cord blood as presented in Table 5. We found a non-significant yet increased risk of SGA status per unit increase in WBSe in maternal blood at delivery and in VC blood. Similarly, we found higher risk for SGA with adjusted models showing significant risk ratios (7–10 % increase per unit increase in VC WBSe).

**Table 5.** Modified Poisson regression analyses (risk ratios) for associations between maternal delivery and venous cord Se biomarkers and adverse birth outcomes (95 % confidence intervals)

Outcome	Se biomarker*	Timepoint	*n*	Unadjusted risk ratio	95 % CI	*P*	*n*	Adjusted risk ratio†	95 % CI	*P*
Small-for-gestational age	WBSe	Delivery	445	1·03	0·96, 1·1	0·4	416	1·04	0·96, 1·1	0·3
	WBSe	Venous cord	441	1·06	0·99, 1·1	0·07	411	1·07	1·0, 1·1	0·05
	Serum Se	Delivery	496	1·02	0·9, 1·1	0·7	486	1·05	0·9, 1·2	0·4
	SEPP1	Delivery	650	0·99	0·85, 1·2	0·9	643	1·03	0·9, 1·2	0·7
	SEPP1	Venous cord	555	0·8	0·6, 1·0	0·08	527	0·7	0·5, 0·9	0·01
Low birth weight	WBSe	Delivery	445	1·05	0·95, 1·2	0·3	416	1·12	1·0, 1·3	0·04
	WBSe	Venous cord	441	1·11	1·0, 1·2	0·02	411	1·08	0·99, 1·2	0·1
	Serum Se	Delivery	496	1·06	0·93, 1·2	0·4	486	1·12	0·97, 1·3	0·1
	SEPP1	Delivery	650	0·99	0·85, 1·2	0·9	643	1·2	1·0, 1·5	0·1
	SEPP1	Venous cord	555	0·8	0·6, 1·0	0·08	527	0·9	0·6, 1·3	0·5
Preterm birth	WBSe	Delivery	467	1·01	0·8, 1·2	0·9	418	1·31	0·9, 1·9	0·2
	WBSe	Venous cord	448	1·13	0·96, 1·3	0·2	413	1·01	0·8, 1·3	0·9
	Serum Se	Delivery	517	1·11	0·9, 1·4	0·3	487	0·99	0·7, 1·4	0·9
	SEPP1	Delivery	677	0·95	0·7, 1·3	0·8	644	1·06	0·67, 1·7	0·8
	SEPP1	Venous cord	562	0·5	0·26, 1·0	0·06	529	1·2	0·5, 3·0	0·7

WBSe, whole blood selenium; SEPP1, selenoprotein P.

* WBSe scaled to represent the change in anthropometric outcome for every 10 µg/l increase in WBSe. Serum Se scaled to represent the change in anthropometric outcome for every 10 µg/l increase in serum Se. SEPP1 scaled to represent the change in anthropometric outcome for every 1000 µg/l increase in SEPP1.

† Models adjusted for infant sex, season of birth, maternal age, maternal education, maternal height, maternal BMI, gravidity, vitamin D treatment group, delivery CRP, asset index quintiles, daily protein intake (kg), smoking and tobacco use during pregnancy.

Associations between LAZ and WBSe in maternal delivery samples were not significant, although we found small negative associations between WBSe in VC blood and LAZ at birth, 12 and 24 months of age. Although effect estimates for associations between WBSe in maternal delivery samples and LAZ were small and in the negative direction, we did not find statistical significance across analyses for WBSe and growth outcomes across age time points. For associations between WBSe in VC blood and LAZ, we found small negative effect estimates across unadjusted models at birth, 1 year and 2 years of age. Although mildly attenuated after adjustment, associations between WBSe in VC blood and LAZ were no longer significant, or in sensitivity analyses, after adjusting for cotinine (online Supplementary Tables 3 and 4). In longitudinal analyses, we found mean LAZ at birth to be −0·12 (SE 0·27), *P* = 0·27, not significantly different from 0. Average change in LAZ as children age, per unit increase in VC WBSe was −0·004 (SE 0·0017), *P* = 0·019 (Akaike Information Criterion (AIC): 7392·3, Bayesian Information Criterion (BIC): 7416·2), which suggests that this form of Se has a marginal and non-significant association with linear growth.

Associations between WAZ and delivery WBSe, although similar in magnitude to those observed for LAZ, were not significant in unadjusted models; however, effect estimates increased marginally, and significance was achieved after adjustment and in sensitivity analyses with cotinine. We found small negative associations between WAZ and WBSe in VC at birth in unadjusted and adjusted models. Overall trends ran in the same direction across unadjusted and adjusted models for other growth outcomes including weight-for-length and head circumference for age z-scores.

#### Serum selenium and growth outcomes

In line with our observations for WBSe, we found similar trends for associations between serum Se at delivery and growth measures, although associations were small and non-significant. Risk ratios were also higher for SGA and LBW per unit increase in serum Se concentrations. Similar to results for WBSe, associations between serum Se and birth weight were also negative and large in magnitude (3–28 g decrease in birth weight per unit increase in serum Se), although these findings were not significant and confidence intervals for effect estimates were wide.

#### Selenoprotein P and growth outcomes

SEPP1, unlike serum and WBSe, showed positive associations with infant birth weight in unadjusted and adjusted models, although results for statistical significance were not consistent across models. Based on our findings, a per unit increase in VC blood SEPP1 was associated with a large increase in birth weight, with estimates ranging between 43 and 82 g between unadjusted and adjusted models. Risk for LBW in our analyses was marginal, with WBSe in VC blood suggesting a protective effect per unit increase in concentrations, although results were non-significant.

We did not see significant or consistent associations between LAZ at any age time point and SEPP1 in maternal delivery plasma samples in unadjusted models. We did observe small positive increases in LAZ scores in adjusted models at birth when examining SEPP1 concentrations in VC blood, and associations were significant after accounting for covariates in adjusted models. Effect estimates suggest a stronger association between VC SEPP1 and LAZ at birth up to 2 years of age. These associations, although similar in magnitude, were not significant at 12 and 24 months of age.

Non-significant positive associations were observed between WAZ and SEPP1 in maternal delivery samples at birth. Significant associations were observed between SEPP1 in VC blood and WAZ. We used uncontrolled longitudinal hierarchical models and found a mean WAZ at birth to be −1·1 (SE 0·1), *P* < 0·001. Average change in WAZ as children age, per unit increase in VC SEPP1 was 0·0001 (SE 0·0001), *P* < 0·05 (AIC: 11 047·9, BIC: 11 083·5). These findings suggest a marginal protective effect of this form of Se on WAZ.

Weight-for-length and head circumference for age z-score were not associated with SEPP1 in maternal delivery and/or VC blood. We did note that the overall risk for SGA and LBW was marginally lower per unit increase in SEPP1 in both maternal delivery and VC blood; however, these relationships were not significant. Across the different biomarkers, we did note shifts in the direction of trends across the different age points at which growth outcomes were measured.

#### Mediation analyses and generalised additive model analyses

**Fig. 2.**
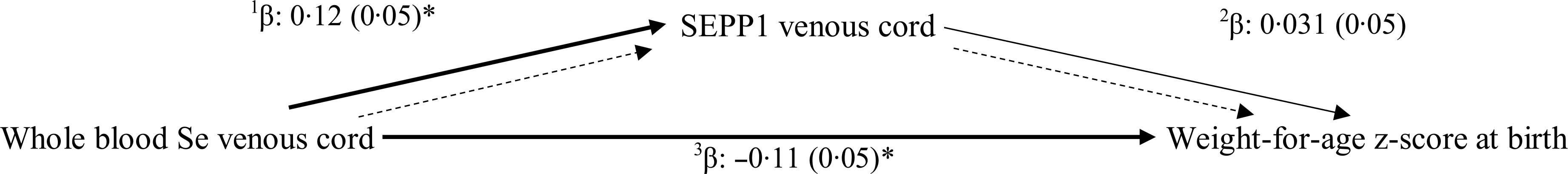
Mediation path analysis exploring associations between WAZ at birth and venous cord Se biomarkers. ^a,b^Unadjusted direct and indirect effects in mediation analysis (*n* 402); total effect: *β*: −0·11 (0·05)*; indirect effect: *β*: 0·004 (0·006). ^c^Fit indices for models: *χ* ^2^:11·3, df: 3, *P*-value: 0·01; CFI: 1·00; TLI: 1·00; SRMR: 0·00; GFI: 1·00; RMSEA: 0·000 (overall model fit is good). *P < 0·05, **P < 0·01.

**Fig. 3.**
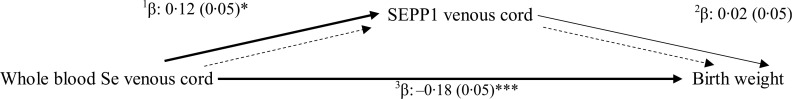
Mediation path analysis exploring associations between birth weight and venous cord Se biomarkers. ^a,b^Unadjusted direct and indirect effects in mediation analysis (*n* 402); total effect: *β*: −0·18 (0·05)***; indirect effect: *β*: 0·003 (0·01). ^c^Fit indices for models: *χ* ^2^:19·5, df: 3, *P*-value: 0·0002; CFI: 1·00; TLI: 1·00; SRMR: 0·00; GFI: 1·00; RMSEA: 0·000 (overall model fit is good). ^d^Unstandardised estimates for path models (to compare *β*-estimates to linear regression models): ^1^ *β*: 0·03 (0·01)*; ^2^ *β*: 13·1 (29·8); ^3^ *β*: −26·9 (7·3)**. *P < 0·05, **P < 0·01, ***P < 0·001, ****P < 0·0001.

**Fig. 4.**
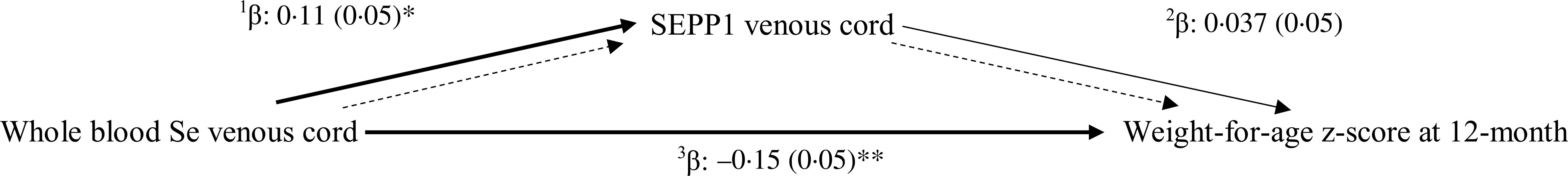
Mediation path analysis exploring associations between WAZ at 12 months of age and venous cord Se biomarkers. ^a,b^Unadjusted direct and indirect effects in mediation analysis (*n* 394); total effect *β*: −0·15 (0·05)**; indirect effect: *β*: 0·004 (0·006). ^c^Fit indices for models: *χ* ^2^:13·7, df: 3, *P*-value: 0·003; CFI: 0·00; TLI: 1·00; SRMR: 0·00; GFI: 1·00; RMSEA: 0·000 (overall model fit is good). *P < 0·05, **P < 0·01.

Results from mediation path analysis are presented in Fig. 2–4. Across path models, the indirect mediation effects, which represent the association of WBSe and weight-related growth outcomes, mediated through SEPP1, were extremely small and non-significant. We found small but significant direct effects between WBSe and SEPP1 in VC blood and growth outcome measures. Overall fit indices for all path models suggest statistical convergence and good fit.

To examine dose–response relationships between LAZ and weight-for-age, we performed GAM analysis with Se biomarkers in maternal delivery specimens and VC blood. Figures are presented in online Supplementary Figures 1 and 2. We found consistent inverse U-shaped associations between LAZ, WAZ and SEPP1 in VC blood and similarly between growth z-scores and serum Se in delivery specimens.

## Discussion

In this study of mother–infant dyads, we found measures of Se status in maternal delivery and VC blood serum, whole blood and plasma SEPP1 to be modestly associated with infant growth outcomes at birth, 12 months and 24 months of age, with variability in the strength and magnitude of associations across biomarkers and age points. Effect sizes in path analyses were negligible and do not suggest any mediation of the association between WBSe and WAZ at birth or 12 months, by VC SEPP1, likely because WBSe and SEPP1 have differing functions and therefore different impacts on weight and act as part of different metabolic pools that do not interact or share common pathways, as described in online Supplementary Fig. 3 ^(30)^.

Several studies have examined the effects of Se in a variety of biological specimen types and their associations with infant growth outcomes^(12,19,31–40)^. A comprehensive comparative list of epidemiological studies is presented in Table 6. Overall concentrations of Se across biomarker types in our study were comparable to those described in the literature.

**Table 6.** Epidemiological studies examining associations between Se status during pregnancy, postpartum and in cord blood and birth/growth outcomes

Authors	Study design and sample size	Measures of Se status	Measures of birth/growth outcomes	Key findings
Bogden *et al*. 2006	CA: (< 37 weeks gestation, *n* 107; sub-set < 32 weeks, *n* 25) CO: *n* 126, ≥ 37 gestation) nested within cohort	Maternal erythrocyte GPx activity (microunits of GPx activity/mg of HgB): 20·9 ± 0·76; 22·4 ± 0·7 Dietary Se intake (24-h recall): 115 ± 9; 93 ± 5 Serum Se (umol/l): 1·37 ± 0·02; 1·34 ± 0·02 Collected in first and second trimesters of pregnancy	Serum Se and birth weight (g): −134 ± 182 (*P* = 0·46) (lowest decile *v*. others, CA, *n* 126) Serum Se and birth weight (g): −270 ± 117 (*P* = 0·02) (lowest decile *v*. others, CO, *n* 107)	Low-normal serum Se measured in first and second trimesters of pregnancy predicts lower birth weight for full-term infants. The risk of LBW was highest in the lowest decile of serum Se, no increase in risk above lowest decile Reduction in birth weight was considerable (–259 g for women in lowest decile of serum Se)
Klapec *et al.* 2008	Cross-sectional mother newborn pairs IUGR – *n* 57; normal – *n* 44	Placental Se levels (µg/g)	Placental level Se (µg/g) and IUGR (median, range): 0·14 (0·10, 0·20), *n* 49; AGA (*n* 36): 0·15 (0·10, 0·24) Se (µg/g) *v*. weight change (g/unit): 6954·92 (508·25, 11 125·61), *P* < 0·05, *R* ^2^0·59	Gestational age, Se content in placenta, maternal BMI and newborn sex were associated with significant contribution to birth weight in AGA infants Maternal serum or placental Se – predict birth weight for full-term infants
Tsuzuki *et al*. 2013	Japan, observational 44 infants 14 premature 2 IUGR	Maternal serum Se at delivery: (pre-term: 79·3 ± 19·3 µg/l *v*. term: 94·1 ± 18·1 µg/l, *P* = 0·032) Cord blood Se at delivery: (pre-mature: 52·8 ± 9·7 µg/l *v*. term: 59·4 ± 10·0 µg/l, *P* = 0·071) Neonatal serum Se at day: (pre-term: 43·3 ± 7·0 µg/l *v*. term: 52·0 ± 8·9 µg/l, *P* = 0·001)	Birth weight (dependent): NeoSe: 17·783 (–5·590, 41·155); *P* = 0·128 MatSe: 10·985 (2·391, 19·578); *P* = 0·015 (maternal (serum Se) predictor of neonatal birth weight independent of maternal nutritional status) UmbSe: −14·524 (–33·134, 4·085); *P* = 0·119	Se status in neonates and mothers is positively associated with birth weight
Mistry *et al*. 2014	126 pregnant adolescent women Prospective observational study – About Teenage Eating Study	(Se µg/l) AGA (*n* 107) à 65·1 (62·7, 67·5) SGA (*n* 19) à 49·4 (45·9, 52·9)	SGA: individualised birth weight ratio < 10th percentile; plasma Se: 60·3 µg/l (White-European); 65·9 µg/l (Afro-Caribbean) OR for SGA: 1·15 (1·07, 1·23); 1·16 (1·08, 1·24), *P* < 0·0001	Odds of SGA are significantly higher among pregnant women with lower plasma Se
Sun *et al*. 2014	209 women, cross-sectional study (19–41 years)	Arithmetic mean (95 % CI) Maternal Se: 156·58 (145·82, 167·34) µg/l Cord Se: 8·71 (7·29, 10·14) µg/g Urine Se: 129·04 (124·30, 133·76) µg/l	Log cord Se and birth weight: 653·80. *P* = 0·01	Cord blood Se is positively associated with birth weight
Nazemi *et al.* 2015	Case: 91 mothers (LBW < 2500 g) Control: 86 mothers (normal birth weight neonates)	Maternal blood Se (µg/ml): CA: 80·69 ± 28; CO: 78·48 ± 25·54 Umbilical blood Se (µg/ml): CA: 77·32 ± 26·12; CO: 73·89 ± 24·37	Maternal blood Se, *P*-value: 0·961 Umbilical cord Se, *P*-value: 0·660	No significant associations found between maternal or umbilical cord Se and birth weight
Bermudez *et al*. 2015	Cross-sectional, *n* 54 paired samples SGA, *n* 11; AGA, *n* 30; Large for Gestational Age, *n* 13	Cord plasma Se (nM/l): 438·4 ± 78·6 Maternal plasma Se (nM/l): 704 ± 199·7 Cord/maternal ratio (mean ± sd): 1·08 ± 3·02	Umbilical cord blood Se and Small for Gestational Age (SGA) (*n* 11): 442·0 ± 79·81 nM/l Umbilical cord blood Se and Average for Gestational Age (AGA) (*n* 30): 439·0 ± 84·83 nM/l Umbilical cord blood Se and Large for Gestationnal Age (LGA) (*n* 13): 433·8 ± 67·37 nM/l Maternal peripheral blood Se and SGA (*n* 8): 680·9 ± 298·6 nM/l Maternal peripheral blood Se and AGA (*n* 25): 712·37 ± 160·2 nM/l Maternal peripheral blood Se and LGA (*n* 13): 701·92 ± 213·91 nM/l	Umbilical cord Se was higher in SGA neonates; however, maternal plasma Se was lower in SGA infants, suggesting preferential transfer to the fetus to sustain growth and development
Hu *et al*. 2016	*n* 81 mother–newborn pairs from 4 cities in China enrolled cross-sectionally	Maternal blood Se (ng/g): 140·8 Newborn cord blood Se (ng/g): 22·0	Birth weight and maternal blood: *β*: 1·0 (–3·6 to 1·6), *P* = 0·459 *β*: 0·5 (–3·2 to 2·2), *P* = 0·707	Positive non-significant associations observed between maternal Se levels and infant birth weight
Kobayashi *et al*. 2019	Japan Environmental and Children’s Study – prospective birth cohort, *n* 15 444 pregnant women	Blood Se: 170·0 (158·0–183·0) ng/g	Compared with infants of mothers with highest blood Se, mothers with lowest blood Se had neither significant increase in birth weight (9 g, 95 % CI −6·25) nor significant odds ratio of SGA (0·903, 95 % CI 0·748, 1·089)	No observed differences between birth weight and SGA and levels of maternal blood Se
Lewandowska *et al*. 2019	Nested prospective cohort of *n* 750 women recruited at 10–14th week of singleton healthy pregnancy in Poland	Mean Serum Se concentration of cohort: 61·95 µg/l (range: 41·14–89·17 µg/l)	SGA: (weight < 10th percentile for GA, sex of newborn in population): *n* 48 AGA: (weight between 10–90th percentile): *n* 192 Q_1_: Se: (41·14–56·60 µg/l); OR for SGA: 2·63 (1·08–6·42); *P* = 0·034 Q_2_: Se: (56·60–61·86 µg/l); OR for SGA: 0·87 (0·36–2·14); *P* = 0·794 Q_3_: Se: (61·86–66·62 µg/l); OR for SGA: 1·42 (0·55–3·66); *P* = 0·472 Q_4_: Se: (66·62–89·17 µg/l); OR for SGA: 1	Lowest concentrations of early pregnancy maternal serum Se associated with higher risk of SGA Threshold found at 56·1 µg/l below which steep increase in SGA risk observed
Sole-Navais *et al*. 2020	MoBa – prospective population-based pregnancy cohort, Norway	WBSe: (*n* 2572 women at 17–18 weeks gestation), median: 102 (89–117) µg/l	Maternal dietary Se intake in first 4–5 months of pregnancy: *β*: 0·027 (95 % CI 0·007, 0·014) Birth weight (g): *β*: −0·47 (–1·29, 0·34), 41·6; *P* = 0·254 Z-scores: *β*: −0·001 (–0·003, 0·001), 0·09, *P* = 0·195 SGA OR: 1·24 (0·53, 2·88), *P* = 0·623	Maternal dietary Se intake in the first 4–5 months of pregnancy was associated with higher birth weight and population-based birth weight z-scores (sd: 14·3 µg/d) of Se intake Negative associations observed between birth weight and maternal whole blood Se, additionally non-significant but higher OR for SGA
Moody *et al*. 2020	Community-based cohort of *n* 100 children 6–59 months	Venous blood Se (median, IQR) (µg/dl): 12·20 (10·69–15·02) Stunted (median, IQR) (µg/dl): 11·53 (9·65–12·13) Not stunted (median, IQR) (µg/dl): 13·05 (10·9–15·49)	*β*: 1·924 (*P* = 0·005)	Significant positive association between Se and HAZ score Se showed a U-shaped toxicity dose–response curve, with negative effects at deficient and toxic levels
Guo *et al*. 2021	Prospective birth cohort study *n* 1931 pregnant women (28–36 gestational weeks)	Maternal serum Se: 136·9 ± 47·9 µg/l	Birth weight (g) (3·4 ± 0·4 kg) *β*: 0·003 (–0·001, 0·006); −0·0002 (–0·0008, 0·00003) Recumbent length (0·1 cm) (49·9 ± 1·9 cm) *β*: 0·004 (–0·003, 0·012); 0·001 (–0·0002, 0·002) Maternal Se levels: ≥ 103·7 µg/l (highest 3 quartiles)	Effect sizes were small and non-significant for the associations between maternal serum Se and birth weight, recumbent length, although associations were positive for Se concentrations in the highest 3 quartiles compared with the lowest Se concentration quartile
Zhang *et al*. 2021	China-Anhui Birth Cohort Study – *n* 3133 mother–infant pairs	Maternal serum Se: 123 µg/l (2·0–440·6)	Birth weight (g): *β*: 0·58 (0·34, 0·82), *P* < 0·0001 Birth length (cm): *β*: 0·001 (0·000, 0·003), *P* = 0·03 Head circumference (cm): *β*: 0·0002 (0·000, 0·001), *P* = 0·61 Chest circumference (cm): *β*: 0·001 (0·000, 0·002), *P* = 0·13	Odds of low birth weight were 3·92 (2·03, 7·57) times higher OR for SGA were 2·77 (1·92, 4·02) times higher among infants with mothers who were Se deficient (< 45 µg/l) OR for LBW were 187 (1·02, 3·45) times higher and OR for SGA were 1·47 (1·07, 2·02) times higher among infants with mother who were Se insufficient (45–94·9 µg/l), all results were statistically significant (*P* < 0·001)

SGA, small-for-gestational age; LBW, low birth weight; IUGR, intra-uterine growth restriction; IQR; inter-quartile range; GPx, glutathione peroxidase.

During pregnancy, Se progressively accumulates in fetal organs^(41)^. Se is stored primarily in the liver of the growing fetus and is likely redistributed after birth, where concentrations in utero remain constant during gestation and decrease in line with stable Se concentration in other organs, during the months following birth^(8)^. Transfer of SEPP1 from maternal blood to fetus is facilitated by Apo-ER2 mediated endocytosis. Selenomethionine containing proteins and SeMet also supply Se to the fetus^(42,43)^.

In a recent review of studies conducted across a variety of different geographies, results of the association between Se and birth and growth outcomes have been mixed, although overall associations suggested a positive and protective effect of this micronutrient (pooled correlation coefficient between VC blood Se and birth weight; *r* 0·08, 95 % CI 0·01, 0·16 and maternal blood Se and birth weight; *r* 0·02 (–0·01, 0·05) on birth weight)^(6)^. In our study, we found a strong negative association between WBSe in VC blood and birth weight (*β*: –2·3, 95 % CI –3·7, –0·98, *P*-value: 0·001); however, the association between SEPP1 in VC and birth weight ran in the opposite direction (*β*: 0·04, 95 % CI –0·01, 0·1, *P*-value: 0·1). Thus, findings negate our hypothesis that SEPP1 may mediate the association between VC WB Se and weight-related outcomes (birth weight, WAZ at birth and at 1 year of age).

The negative association between WBSe in VC blood and birth weight may be a result of high Se exposure in utero. This can lead to oxidative stress, which in turn causes hypoxia and can impact fetal growth by reducing the distribution of oxygen in the placenta^(44,45)^. It is important to note that Se concentrations at levels classified as toxic were not detected in any of our specimens (> 400 µg/l)^(46)^. Non-selenoprotein bound Se concentrations are known to be higher in the placentas of women who experience intra-uterine growth restriction and in mothers of preterm infants^(9)^. These findings add further credence to our observations around the negative associations between WBSe and birth weight^(45)^.

The protective antioxidant effects of Se bound to selenoproteins including SEPP1 may work to increase birth weight during gestation. This may explain the positive associations with birth weight as observed in our study, given the known antioxidant properties of selenoproteins, which can protect the fetus from free radicals in utero, modulate inflammation and protect biological membranes and DNA during early stages of embryo development^(6)^. As expected, a plateauing effect was noted in our results, for the association between WBSe and functional selenoprotein, SEPP1 in VC blood at WBSe concentrations of approximately 160 µg/l; however, no distinct plateauing was observed for associations with delivery biomarkers.

Mixed findings about the effects of varied biomarkers of Se status on placental development and growth-related outcomes, postpartum, may be explained by differences in bioavailability of organic and inorganic forms of the micronutrient^(46)^. This is important because absorption of these forms of Se, once in the bloodstream, can impact distribution, partitioning and potential toxicity at high concentrations^(46)^.

The primary mechanisms whereby Se has been associated with adverse growth outcomes include its essential role in antioxidant defence and its role in mediating oxidative stress, which could impact peri-natal morbidity and mortality^(9)^. This in turn may impact risk of outcomes such as miscarriage, pre-eclampsia, gestational diabetes and intra-uterine growth restriction^(9)^.

Low Se status, particularly in women, can impact the risk of PTB^(9)^. Our results are in contrast to these findings however, where we found higher odds of PTB per unit increase in maternal delivery whole blood and serum Se and WBSe in VC blood^(9)^. These findings were not significant after accounting for confounding variables. Associations have been noted between lower serum Se and PTB (OR: 2·0, 95 % CI 1·19, 3·47)^(47)^. On the other hand, the risk of PTB was lower per unit increase in SEPP1 in maternal delivery and VC blood specimens, although our results did not approach statistical significance.

Mechanistically, Se and PTB are linked because of the role this micronutrient plays in the body’s inflammatory response which can in turn cause ruptures to the fetal membrane^(47)^. Serum Se concentrations that surpass 80·5–86·9 µg/L can cause an attenuation in the odds of PTB^(47)^. We noted similar findings in our study, where we saw an attenuation in OR for the highest quintile of serum Se concentrations in our sample (OR: 1·3, 95 % CI 0·7, 2·4) compared with the lowest, and a decreasing trend in OR by increasing quintiles of serum Se.

Women at increased risk of miscarriage may also have lower serum Se concentrations, particularly during the first trimester of pregnancy^(48)^. Furthermore, cord blood Se concentrations are known to be significantly lower in premature infants when compared with neonates born at term^(49)^.

Similar to other birth-related outcomes, an increased risk for SGA has also been observed in relation to Se, likely driven by mechanisms associated with oxidative stress^(50)^. We found a per unit increase in concentrations of several Se biomarkers to result in a small and non-statistically significant increase in the risk of SGA. It is likely that inflammation plays a role in our findings, given that median concentrations of C-reactive protein at delivery were 9 mg/l, which are higher than clinically defined thresholds for this acute phase protein (< 5 mg/l)^(51)^. We have therefore adjusted our models to account for potential confounding. We noted an attenuation in the odds of LBW across quintiles of maternal serum Se at delivery, which suggest that higher concentrations of Se are associated with lower odds of LBW (OR: 0·89, 95 % CI 0·6, 1·4), which is consistent with other studies^(39)^.

In our study, we found very small negative associations between WBSe in VC blood and LAZ across age time points, in unadjusted and adjusted linear models, although other Se biomarkers were not associated with LAZ. We observed small effect sizes that were not significant for associations between serum Se at delivery and LAZ. This was in line with the literature for maternal serum Se^(19,39)^.

Linear growth has been implicated as a potential outcome of interest in relation to the role of Se in bone growth. Kashin–Beck disease is a chronic osteochondropathy known to result from inherited genetic polymorphisms in Se-related genes which impair collagen synthesis^(52,53)^. The syndrome is endemic to regions with low soil Se concentrations^(52)^. A key feature of the condition is necrosis of chondrocytes in the growth plate of the bone and within the articular surface, which results in growth retardation and osteoarthritis^(54,55)^. In this condition, Se status may modulate disease susceptibility because of its essential antioxidant activities. Three major environmental hypotheses have been postulated as part of the aetiology of the condition and include soil Se deficiency, contamination of cereals by mycotoxigenic fungi and high humic acid concentrations in the drinking water supply in endemic areas including China and Tibet^(56)^. In a clinical study conducted in Lhasa, Tibet, where Kashin–Beck disease is endemic and Se deficiency is common, authors found serum Se concentrations to be below detectable levels (< 5 µg/l) in 38 % of the study sample (*n* 521 subjects). Geometric mean concentrations of serum Se in this study sample for subjects who had Kashin–Beck disease were as low as 10·3 (5·1–20·8) µg/l. These values are significantly below those observed in our study from Bangladesh, where we found mean concentrations of maternal serum Se to be 69 (sd 13) µg/l at delivery. These findings suggest that severe Se deficiency may not be a problem in this Bangladeshi population (83·4 % of our sample was < 80 µg/l for serum Se at delivery and only 0·2 % were Se deficient (< 30 µg/l)).

It is important to note that effect estimates for other growth outcomes in our study were negligible in magnitude and therefore do not suggest that Se biomarkers measured in our study play a major role in birth and growth outcomes in this cohort of mother–infant pairs. We did however find meaningful and substantial effect sizes for the associations between birth weight and biomarkers of Se status. These associations should be investigated further given that different Se biomarkers may act differently on mechanisms of weight gain at birth and in early infancy.

Biomonitoring equivalents for toxic concentrations of Se in a variety of body fluids have been calibrated using various regulatory limits set by the Institute of Medicine (upper limits), USEPA (reference dose) and ATSDR chronic (minimum risk level) and were established using Chinese cohort data to define guideline levels to protect against selenosis^(46)^. These values range between 400 and 480 µg/l in whole blood and 180 and 230 µg/l in plasma^(46)^. In our sample, we found maximum levels of WBSe to range between 190·6 and 233·4 µg/l (∼50 % less than WB ranges) in delivery and VC blood and 110·3 µg/l (∼ 60 % less than WB ranges) in serum specimens, which do not suggest risk for toxicity due to selenosis in this population.

Evidence exists to suggest that Se has a U-shaped dose–response toxicity curve, with negative health impacts experienced due to deficiency and excess as is evidenced in our dose–response analyses as well^(57,58)^. Se deficiency may contribute to inflammation and pregnancy-related complications, via pathways associated with oxidative stress and may also be associated with growth retardation^(59)^. At high concentrations, Se can become a pro-oxidant and cause cellular damage due to oxidative stress^(59)^. In conclusion, we have found that Se may play an important role in placental health and the growth and development of infants and young children in the peri-natal and postpartum period, up to 2 years of age. Future studies should examine, in more detail, the role of Se in linear growth over the infant and young child life course, particularly in relation to its important known functions in placental preservation and birth weight.

## Supplementary material

For supplementary material accompanying this paper visit https://doi.org/10.1017/S0007114523000739. click here to view supplementary material
