# Supplementary material for: Associations between maternal and infant selenium status and child growth in a birth cohort from Dhaka, Bangladesh
Source: Br J Nutr. 2023 Mar 22;130(9):1558–72. doi: 10.1017/S0007114523000739 (PMC10551473; doi:10.1017/S0007114523000739)
Supplement: Supplementary file 1 [file S0007114523000739sup001.pdf]

Supplementary Figure 1: General additive models for associations between length-for-age z-scores and selenium biomarkers at delivery and in venous cord blood

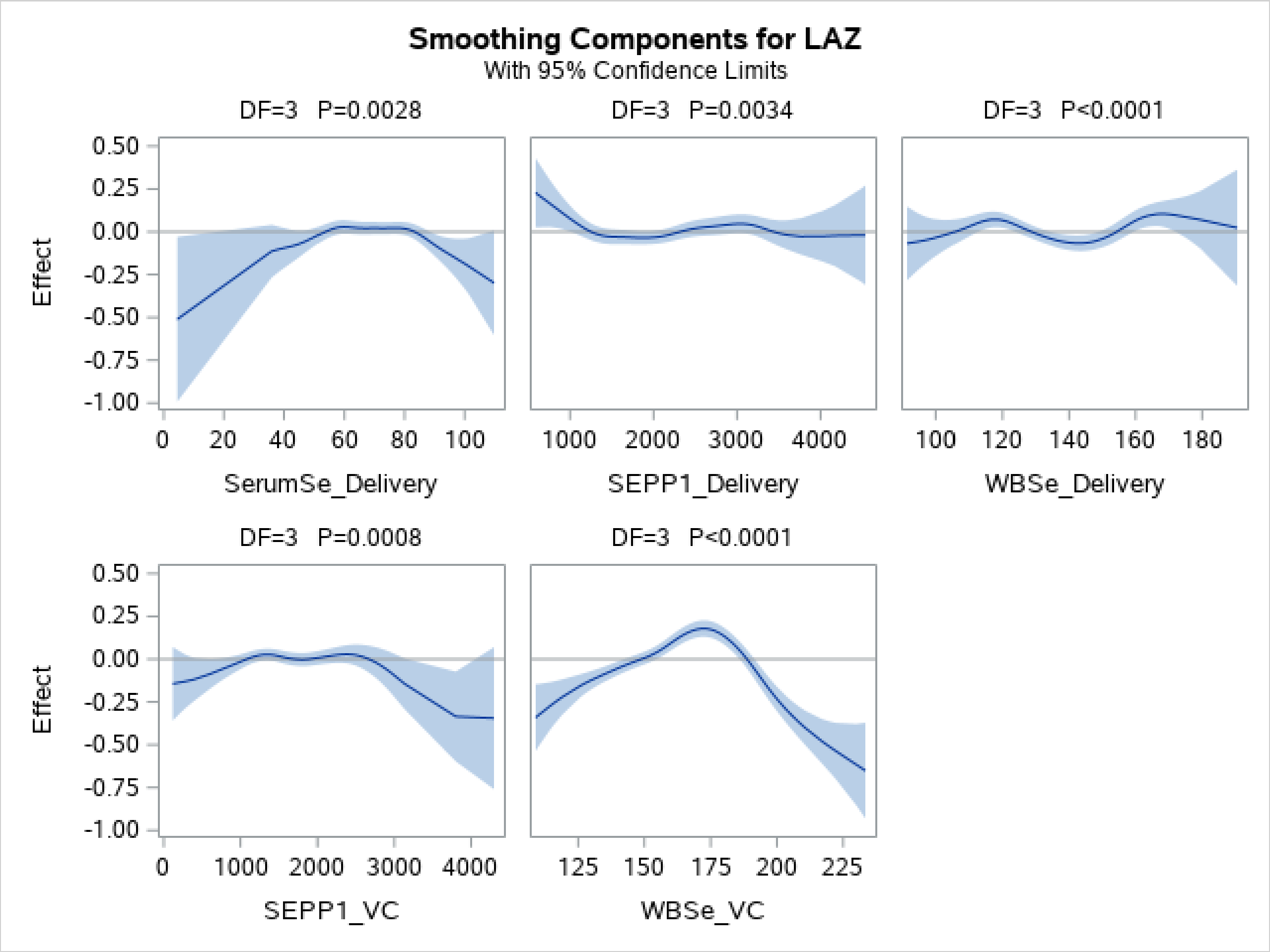

Supplementary Figure 2: General additive models for associations between weight-for-age z-scores and selenium biomarkers at delivery and in venous cord blood

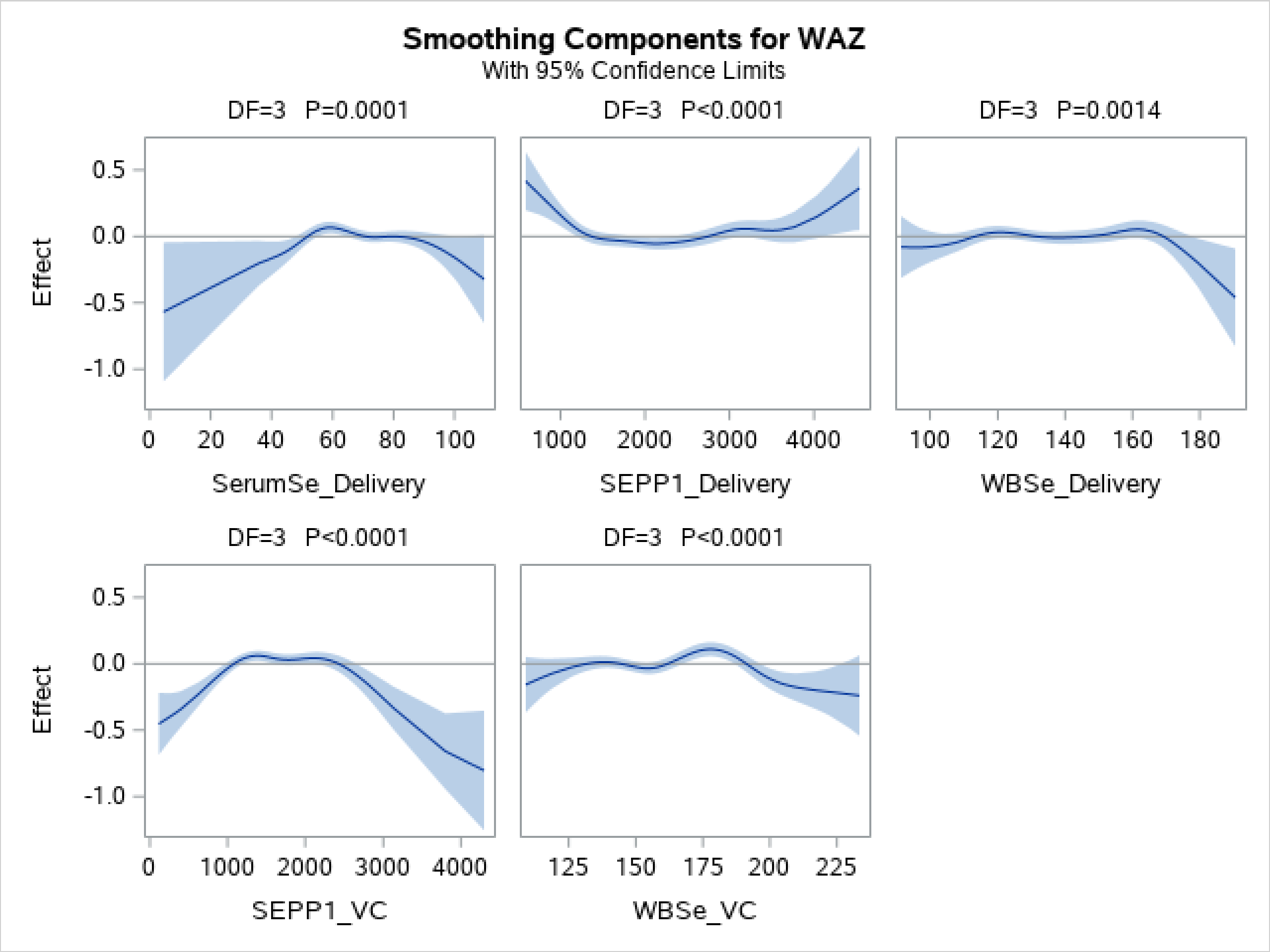

Supplementary Figure 3: Mechanisms of Selenium transport from placenta to fetus during pregnancy and pathways of metabolism for different forms of selenium in humans

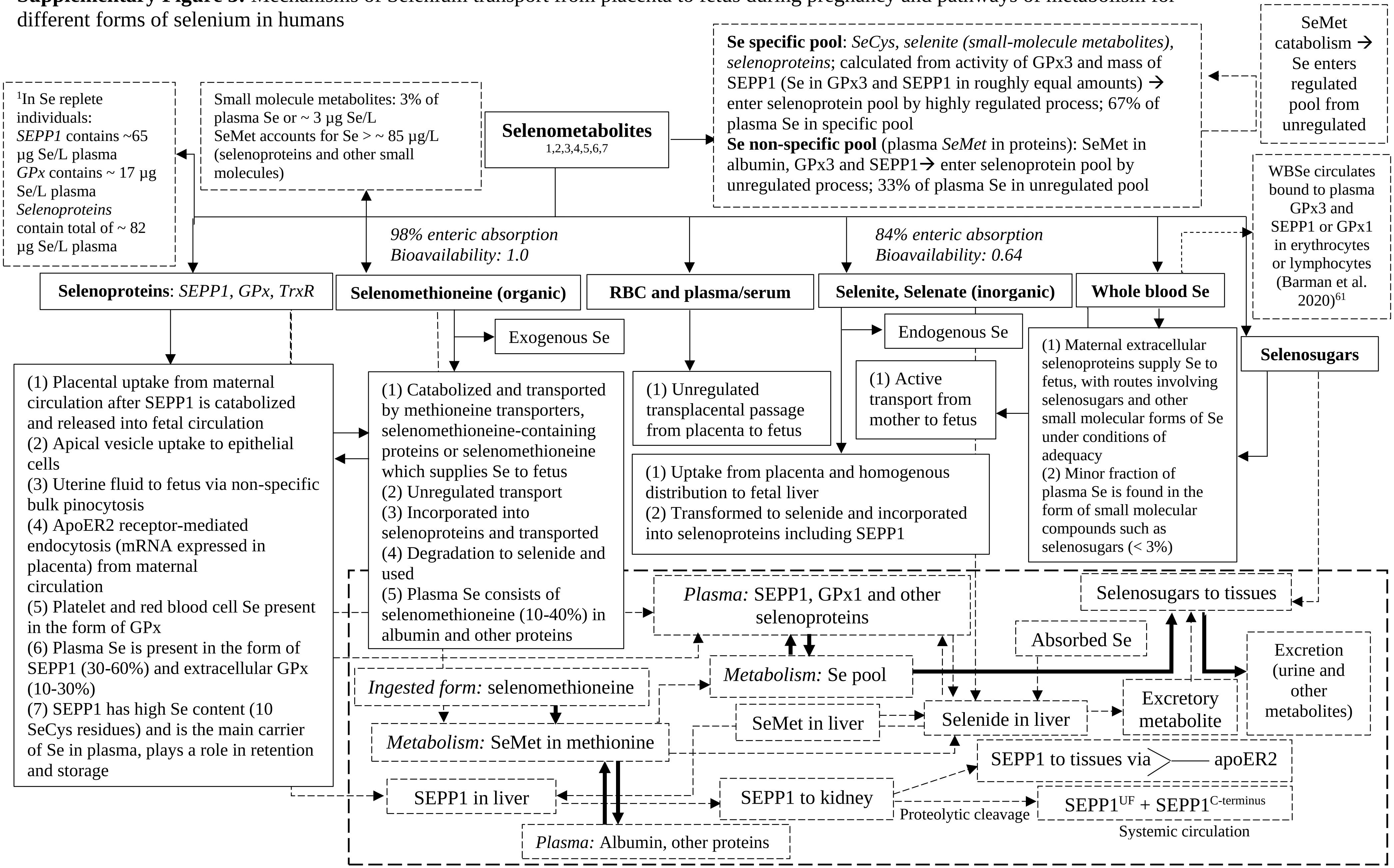

——— Placental transport of selenometabolites in humans during pregnancy

- - - - Selenium metabolism and transport in the body (not exclusive to pregnancy) across assorted selenometabolites (adapted from Burk et al., 2015)[45]

━━━ Ingested Se and plasma forms of Se (SeMet in Met pool metabolized via trans-sulfuration pathway and SeCys *β-lysase* → metabolically active Se in the Se pool)

Selenosugar (1β-methylseleno-*N*-acetyl-D-galactosamine) is filtered into urine by the kidney, it does not accumulate in plasma

Adapted from: Burk et al., (2013)[62]; Chen et al., (2014)[63]; Santos et al., (2017)[64]; Anan et al., (2008)[65]; Al-Saleh et al., (2015)[66]; Burk et al., (2015)[45]; EFSA (2014)[8]; Burk et al., (2006)[67]

Supplementary Table 1: Selenoprotein concentrations in mid-gestation and delivery specimens in the growth study

	Maternal mid-gestation (17-24 weeks)		Maternal delivery	
**Biomarkers**	**GPx**	**TrxR**	**GPx**	**TrxR**
N[1]	*201*	*201*	*145*	*144*
**Selenium biomarker concentration (μg/L)**, median (IQR)	135.3 (32.3)	4.8 (2.8)	138.3 (36.8)	7.3 (8.1)
Gestational age at birth (in weeks), median (IQR)	-	-	38.9 (1.9)	38.9 (1.9)
Birth weight (g), mean (SD)	-	-	2647.5 (470.0)	2653.8 (481.3)

[1]Sample sizes exclude observations where measures of selenoprotein & birth weight are not available

Supplementary Table 2: Linear associations between selenoproteins – GPx and TrxR and birth weight

Outcome	Se Biomarker	Timepoint	N	Unadjusted β (95% CI)	p-value	N	Adjusted β[2] (95% CI)	p-value	N	Adjusted β[2] (95% CI)	p-value
									**Cotinine Sensitivity Analysis**		
Birth weight[1]	GPx	Mid-gestation	145	1.1 (-0.2, 2.3)	0.09	**140**	**1.3 (0.1, 2.5)**	**0.04**	88	1.0 (-0.7, 2.6)	0.2
		Delivery	145	-0.7 (-1.8, 0.4)	0.2	**141**	**-1.1 (-2.1, -0.07)**	**0.04**	90	-0.6 (-2.0, 0.8)	0.4
	TrxR	Mid-gestation	145	4.6 (-8.2, 17.5)	0.5	140	10.6 (-1.6, 22.8)	0.09	88	15.6 (-3.4, 34.6)	0.1
		Delivery	144	-0.1 (-6.9, 6.7)	0.9	140	0.09 (-7.0, 7.1)	0.9	89	1.6 (-7.5, 10.6)	0.7

[1]No scaling applied to GPx and TrxR biomarkers

[2]Models adjusted for infant sex, gestational age at birth, season of birth, maternal age, maternal education, maternal height, maternal BMI, gravidity, vitamin D treatment group, delivery CRP, asset index quintiles, daily protein intake (kg), smoking and tobacco use during pregnancy, cotinine included in sensitivity analysis

Supplementary Table 3: Linear regression sensitivity analyses for associations between maternal delivery and venous cord Selenium biomarkers and growth outcomes adjusting for urinary cotinine

Outcome	Se Biomarker[1]	Timepoint	N	Adjusted β[2,3] (95% CI)	p-value
	**Cotinine Sensitivity Analysis**				
Birth weight	WBSe Del	Birth	**307**	**-30 (-50.1, -9.8)**	**0.004**
	WBSe VC	Birth	**247**	**-27.2 (-44.4, -10.0)**	**0.002**
	Serum Se Del	Birth	280	-27.7 (-58.3, 2.8)	0.08
	SEPP1 Del	Birth	294	4.2 (-48.2, 56.6)	0.9
	SEPP1 VC	Birth	**249**	**79.0 (9.5, 148.4)**	**0.03**
LAZ	WBSe Del	Birth	303	-0.06 (-0.1, 0.007)	0.08
		12-months	366	-0.04 (-0.09, 0.02)	0.2
		24-months	335	-0.01 (-0.07, 0.04)	0.2
LAZ	WBSe VC	Birth	**246**	**-0.08 (-0.1, -0.02)**	**0.006**
		12-months	**258**	**-0.06 (-0.1, -0.008)**	**0.02**
		24-months	238	-0.04 (-0.09, 0.02)	0.2
LAZ	Serum Se Del	Birth	277	-0.07 (-0.2, 0.04)	0.2
		12-months	332	-0.05 (-0.1, 0.03)	0.2
		24-months	303	-0.05 (-0.1, 0.03)	0.3
LAZ	SEPP1 Del	Birth	291	0.02 (-0.2, 0.2)	0.8
		12-months	345	-0.006 (-0.1, 0.1)	0.9
		24-months	318	-0.04 (-0.2, 0.9)	0.6
LAZ	SEPP1 VC	Birth	**247**	**0.3 (0.05, 0.5)**	**0.02**
		12-months	260	-0.02 (-0.2, 0.2)	0.8
		24-months	241	0.06 (-0.2, 0.3)	0.6
WAZ	WBSe Del	Birth	**307**	**-0.07 (-0.1, -0.02)**	**0.004**
		12-months	**366**	**-0.06 (-0.1, 0.001)**	**0.05**
		24-months	335	-0.01 (-0.08, 0.05)	0.7
WAZ	WBSe VC	Birth	**247**	**-0.07 (-0.1, -0.03)**	**0.002**
		12-months	**258**	**-0.06 (-0.1, -0.008)**	**0.03**
		24-months	238	-0.03 (-0.09, 0.03)	0.3
WAZ	Serum Se Del	Birth	280	-0.07 (-0.1, 0.008)	0.08

		12-months	332	-0.06 (-0.1, 0.03)	0.2
		24-months	303	-0.04 (-0.1, 0.06)	0.4
WAZ	SEPP1 Del	Birth	294	0.01 (-0.1, 0.1)	0.9
		12-months	345	-0.06 (-0.2, 0.08)	0.4
		24-months	318	-0.1 (-0.3, 0.06)	0.2
WAZ	SEPP1 VC	Birth	**527**	**0.2 (0.07, 0.3)**	**0.003**
		12-months	260	-0.1 (-0.3, 0.1)	0.4
		24-months	241	-0.04 (-0.3, 0.2)	0.8
WFL	WBSe Del	Birth	269	-0.04 (-0.1, 0.03)	0.2
		12-months	366	-0.05 (-0.1, 0.005)	0.07
		24-months	335	-0.01 (-0.08, 0.06)	0.8
WFL	WBSe VC	Birth	219	-0.05 (-0.1, 0.01)	0.1
		12-months	258	-0.05 (-0.1, 0.009)	0.1
		24-months	238	-0.02 (-0.08, 0.05)	0.6
WFL	Serum Se	Birth	247	-0.01 (-0.1, 0.09)	0.8
		12-months	332	-0.05 (-0.1, 0.04)	0.3
		24-months	303	-0.02 (-0.1, 0.08)	0.7
WFL	SEPP1 Del	Birth	260	0.06 (-0.1, 0.2)	0.5
		12-months	345	-0.07 (-0.2, 0.07)	0.3
		24-months	318	-0.1 (-0.3, 0.05)	0.2
WFL	SEPP1 VC	Birth	218	0.04 (-0.2, 0.3)	0.7
		12-months	260	-0.1 (-0.4, 0.1)	0.3
		24-months	241	-0.09 (-0.4, 0.2)	0.5
HCAZ	WBSe Del	Birth	306	-0.05 (-0.1, 0.01)	0.1
		12-months	363	-0.03 (-0.08, 0.03)	0.3
		24-months	333	-0.0005 (-0.06, 0.06)	0.9
HCAZ	WBSe VC	Birth	247	-0.02 (-0.08, 0.03)	0.4
		12-months	256	-0.02 (-0.07, 0.03)	0.4
		24-months	238	-0.01 (-0.06, 0.03)	0.6
HCAZ	Serum Se Del	Birth	279	-0.08 (-0.2, 0.01)	0.08
		12-months	329	0.02 (-0.07, 0.1)	0.7
		24-months	302	-0.00002 (-0.9, 0.09)	0.9
HCAZ	SEPP1 Del	Birth	293	-0.1 (-0.3, 0.04)	0.1

		12-months	342	-0.06 (-0.2, 0.07)	0.4
		24-months	**317**	**-0.2 (-0.3, -0.02)**	**0.03**
HCAZ	SEPP1 VC	Birth	249	0.04 (-0.2, 0.3)	0.7
		12-months	257	-0.1 (-0.3, 0.1)	0.4
		24-months	241	-0.2 (-0.4, 0.007)	0.06

[1]Whole blood selenium (WBSe) scaled to represent the change in anthropometric outcome for every 10 µg/L increase in WBSe
[1]Serum selenium scaled to represent the change in anthropometric outcome for every 10 µg/L increase in serum Se
[1]SEPP1 scaled to represent the change in anthropometric outcome for every 1000 µg/L increase in SEPP1
[2]Models adjusted for infant sex, gestational age at birth, season of birth, maternal age, maternal education, maternal height, maternal BMI, gravidity, vitamin D treatment group, delivery CRP, asset index quintiles, daily protein intake (kg), smoking and tobacco use during pregnancy and urinary cotinine in sensitivity analysis
[3]No multi-collinearity problem was detected in models – assessment was conducted after generating directed acyclic graphs (DAG) based on conceptual frameworks developed *a priori*

Supplementary Table 4: Modified Poisson Regression sensitivity analyses for associations between maternal delivery and venous cord Selenium biomarker and birth outcomes adjusting for cotinine analysis

Outcome	Se Biomarker	Timepoint	N	Adjusted Risk Ratio (95% CI)	p-value
	**Cotinine Sensitivity Analysis**				
Small-for-gestational age (SGA)	WBSe Del	Delivery	307	1.06 (0.96, 1.2)	0.3
	WBSe VC	Venous Cord	247	1.10 (1.0, 1.2)	0.02
	Serum Se Del	Delivery	280	1.11 (0.96, 1.3)	0.2
	SEPP1 Del	Delivery	294	0.98 (0.75, 1.3)	0.9
	SEPP1 VC	Venous Cord	249	0.8 (0.6, 1.2)	0.3
Low birth weight (LBW)	WBSe Del	Delivery	307	1.15 (1.0, 1.3)	0.03
	WBSe VC	Venous Cord	247	1.12 (1.0, 1.2)	0.06
	Serum Se Del	Delivery	280	1.18 (0.97, 1.4)	0.1
	SEPP1 Del	Delivery	294	1.12 (0.78, 1.6)	0.5
	SEPP1 VC	Venous Cord	249	0.9 (0.6, 1.5)	0.8
Preterm birth (PTB)	WBSe Del	Delivery	308	1.42 (0.9, 2.1)	0.08
	WBSe VC	Venous Cord	248	0.99 (0.7, 1.5)	0.9
	Serum Se Del	Delivery	281	1.65 (0.96, 2.8)	0.07
	SEPP1 Del	Delivery	295	1.64 (0.63, 4.3)	0.3
	SEPP1 VC	Venous Cord	250	1.4 (0.3, 7.3)	0.7

[1]Whole blood selenium (WBSe) scaled to represent the change in anthropometric outcome for every 10 µg/L increase in WBSe
[1]Serum selenium scaled to represent the change in anthropometric outcome for every 10 µg/L increase in serum Se
[1]SEPP1 scaled to represent the change in anthropometric outcome for every 1000 µg/L increase in SEPP1
[2]Models adjusted for infant sex, gestational age at birth, season of birth, maternal age, maternal education, maternal height, maternal BMI, gravidity, vitamin D treatment group, delivery CRP, asset index quintiles, daily protein intake (kg), smoking and tobacco use during pregnancy and urinary cotinine in sensitivity analysis
[3]No multi-collinearity problem was detected in models – assessment was conducted after generating directed acyclic graphs (DAG) based on conceptual frameworks developed *a priori*
